# Heterotopic Porcine Cardiac Xenotransplantation in the Intra-Abdominal Position in a Non-Human Primate Model

**DOI:** 10.1038/s41598-020-66430-x

**Published:** 2020-07-01

**Authors:** Corbin E. Goerlich, Laura DiChiacchio, Tianshu Zhang, Avneesh K. Singh, Billeta Lewis, Ivan Tatarov, Alena Hershfeld, Faith Sentz, David Ayares, Philip Corcoran, Keith Horvath, Muhammad M. Mohiuddin

**Affiliations:** 1 University of Maryland School of Medicine, Department of Surgery, Baltimore, MD USA; 2 Johns Hopkins School of Medicine, Department of Surgery, Baltimore, MD USA; 3 Johns Hopkins Suburban Hospital, Bethesda, MD USA; 4 Association of American Medical Colleges, Washington, DC USA; 5 Revivicor, Inc, Blacksburg, VA USA

**Keywords:** Translational research, Heart failure, Preclinical research

## Abstract

Heterotopic cardiac transplantation in the intra-abdominal position in a large animal model has been essential in the progression of the field of cardiac transplantation. Our group has over 10 years of experience in cardiac xenotransplantation with pig to baboon models, the longest xenograft of which survived over 900 days, with rejection only after reducing immunosuppression. This article aims to clarify our approach to this model in order to allow others to share success in long-term survival. Here, we demonstrate the approach to implantation of a cardiac graft into the intra-abdominal position in a baboon recipient for the study of transplantation and briefly highlight our model’s ability to provide insight into not only xenotransplantation but across disciplines. We include details that have provided us with consistent success in this model; performance of the anastomoses, de-airing of the graft, implantation of a long-term telemetry device for invasive graft monitoring, and ideal geometric positioning of the heart and telemetry device in the limited space of the recipient abdomen. We additionally detail surveillance techniques to assess long-term graft function.

## Introduction

Heart failure is an ever-growing disease with a growing number of patients in end-stage disease, requiring heart transplantation. However, there is a shortage of organs available to replace failing hearts in these patients. Organs from other species, such as swine, have been proposed to meet the demand in these situations as they are anatomically similar to human hearts, genetically manipulatable, have short breeding cycles and are readily available. However, the arduous immunologic barriers between cross-species transplantation has limited its immediate use.

Heterotopic cardiac transplantation in the intra-abdominal position in a large animal model has been essential in the progression of the field of cardiac transplantation. Our group has over 10 years of experience in cardiac xenotransplantation with pig to baboon models, the longest xenograft of which survived over 900 days, with rejection only after reducing immunosuppression^1^. This abdominal model facilitates immunologic monitoring through the period from implantation until graft rejection at reduced cost and complexity compared to orthotopic (life-supporting) models, with the additional advantage that, since the native heart remains in place, rejection of the heterotopic xenograft does not result in primary hemodynamic compromise and/or death.

Here, we demonstrate the approach to implantation of a cardiac graft into the intra-abdominal position in a baboon recipient for the study of transplantation and briefly highlight our model’s ability to provide insight into not only xenotransplantation but across disciplines. We include details that have provided us with consistent success in this model; performance of the anastomoses, de-airing of the graft, implantation of a long-term telemetry device for invasive graft monitoring, and ideal geometric positioning of the heart and telemetry device in the limited space of the recipient abdomen. We additionally detail surveillance techniques to assess long-term graft function.

This heterotopic model, namely that it provides a readily reproducible method for long-term and whole-organ cardiac perfusion without compromising the recipient, should be seen as a standard model for testing iterative improvements in immunosuppression regimens and xenograft genetic manipulations for the further enhancements of cardiac xenotransplantation and allotransplantation at large. While we describe this in the context of transplantation from our extensive experience using this model, considerations are otherwise similar in any other large animal model. As this model uniquely provides *in vivo* assessment of whole organ function without compromising host physiology, it can be used for assessing cardiac physiology across disciplines, where other models have failed or are limited.

## Materials

Specific pathogen-free (SPF) baboons of either sex weighing 15–30 kg (2–3 years of age) from Oklahoma University of Health Sciences (Norman, OK) were housed in a clean pathogen-free facility and were used as recipients. 6 to 8 week-old genetically modified swine of either sex, with an established genetic backbone known to produce prolonged xenograft survival, alpha 1–3 galactosyltransferase gene knockout (GTKO) and overexpression of human CD46 (hCD46) and thrombomodulin (hTBM), GTKO.hCD46.hTBM, were used as donors (Revivicor Inc., Blacksburg, VA) as our standard donor^1^. However, we have also demonstrated success in pigs that additionally express human transgenes for thromboregulation (endothelial protein C receptor, tissue factor pathway inhibitor), complement inhibition (decay accelerating factor), and cellular immune suppression (hCD39, hCD47). SPF baboons were selected for low non-gal antibody titers as previously published^2^. Critical materials are listed in Table 1 and the immunosuppression regimen has been previously described^1,3–5^.

**Table 1:** Additional information on critical materials for heterotopic cardiac transplantation: while these are suggestions based on materials we have used, there are likely other suitable alternatives.

Heterotopic Cardiac Xenotransplantation-Important Materials		
Name	Manufacterer	Reference #
9 Fr Cardioplegia Aortic Root Cannula	Medtronic (USA)	20012
Bladder Irrigation Tubing for Cardioplegia	Baxter (USA)	2C4041
Extension IV Tubing Set	icumedical (USA)	12656-28
Plegisol	Hospira Inc. (USA)	409796905
Data Sciences International (DSI) Telemetry Device L21	DSI (USA)	DSI L21
10 Fr Hickmann Tunneled Triple Lumen Catheter	BARD (USA)	606560

## Methods

All procedures described here have been approved by the Institutional Animal Care and Use Committee (IACUC) at the University of Maryland School of Medicine. All methods were carried out in accordance with relevant guidelines and regulations.

### Heterotopic cardiac transplantation technique

**Figure 1:**
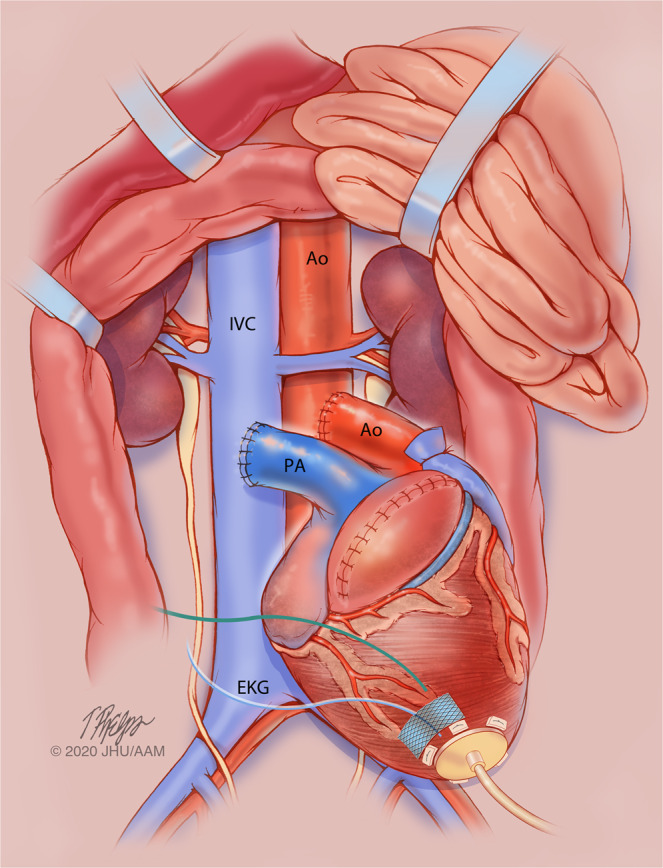
Intraabdominal placement of a cardiac xenograft with pressure telemetry monitor in the apex. IVC-inferior vena cava, Ao-Aorta, PA-pulmonary artery, EKG-electrocardiogram leads from telemetry device. Of note, whereas the telemetry device depicted here only has one pressure sensor and it is placed in the left ventricle at the apex, the pressure can be placed in the right atrium, pulmonary artery or aorta as well, depending on which hemodynamic parameters of interest are to be studied. Image Copyright: Tim Phelps JHU/AAMM, 2020.

The intra-abdominal heterotopic model (HHTx) is a two-anastomosis system utilizing arterial supply from the infrarenal aorta of the recipient baboon to perfuse the coronaries of the donor heart with drainage of the cardiac graft through the donor pulmonary artery remnant anastomosed to the intra-abdominal inferior vena cava of the baboon recipient (Fig. 1). A list of critical materials is provided in Table 1. A brief description is provided followed by step-by-step instructions for performing HHTx. A supplemental video is also provided (supplementary video 1).

**Figure 2:**
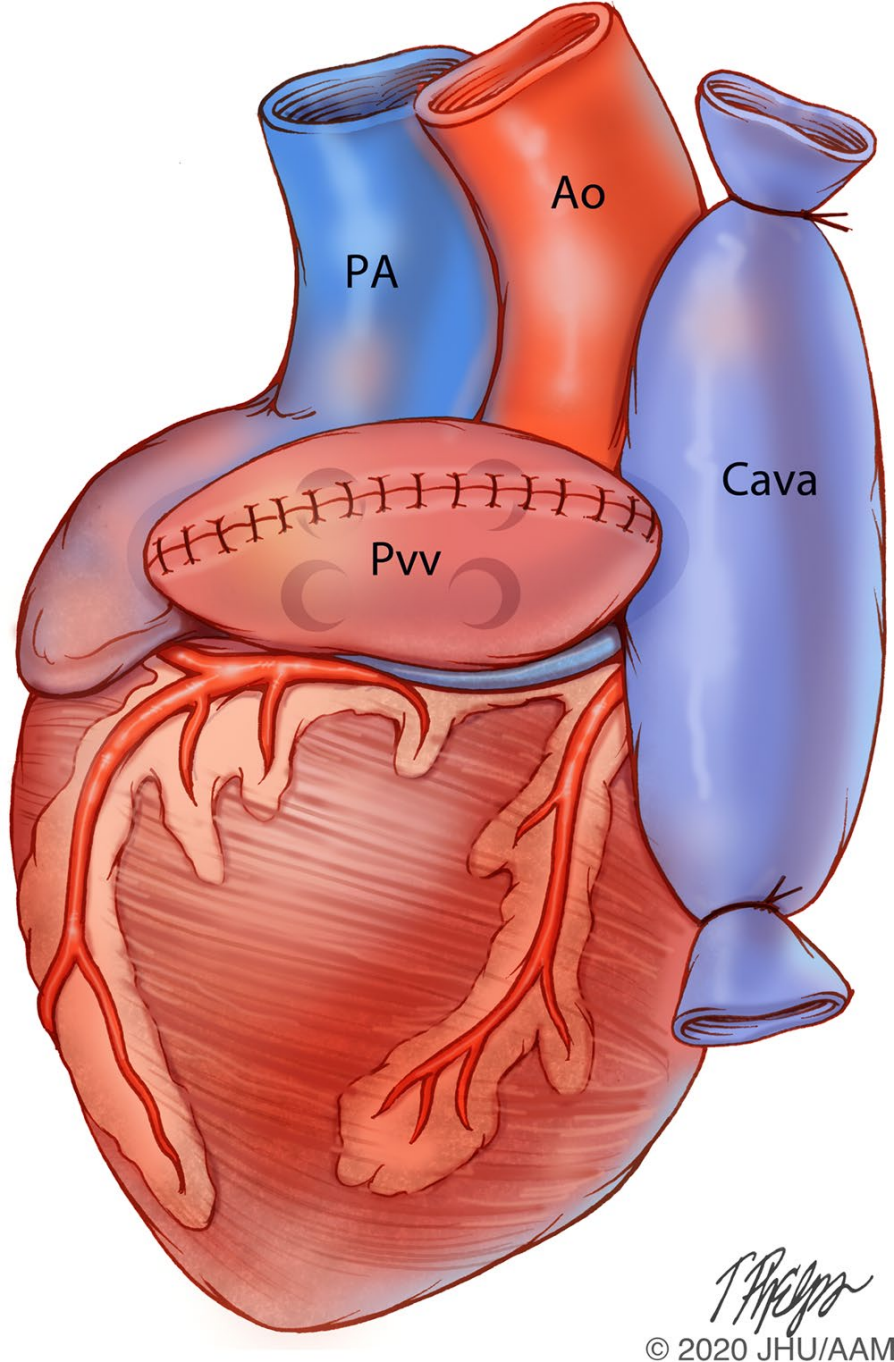
Backtable preparation of the heart for transplantation. Ao-ascending aorta, PA-pulmonary artery, Pvv-Pulmonary vein common channel, Cava-superior and inferior vena caval junction. Image Copyright: Tim Phelps JHU/AAMM, 2020.

Briefly, the cardiac donor is prepped and draped sterilely, and a midline sternotomy is performed. Pericardium is opened and major vessels are isolated. Silk ties are placed around both superior (SVC) and inferior (IVC) vena cavae. Cold blood cardioplegia is administered through a 9 Fr aortic root canula after ligating the SVC and applying a vascular cross clamp on the aorta. The heart is decompressed by venting the IVC and left atrium or pulmonary vein and cardiectomy is performed. The heart is placed on ice during backtable preparation. The IVC is ligated and pulmonary vein common channel is created and over sewn (Fig. 2). The cardiac graft is now readily for transplantation into the abdomen.

**Figure 3:**
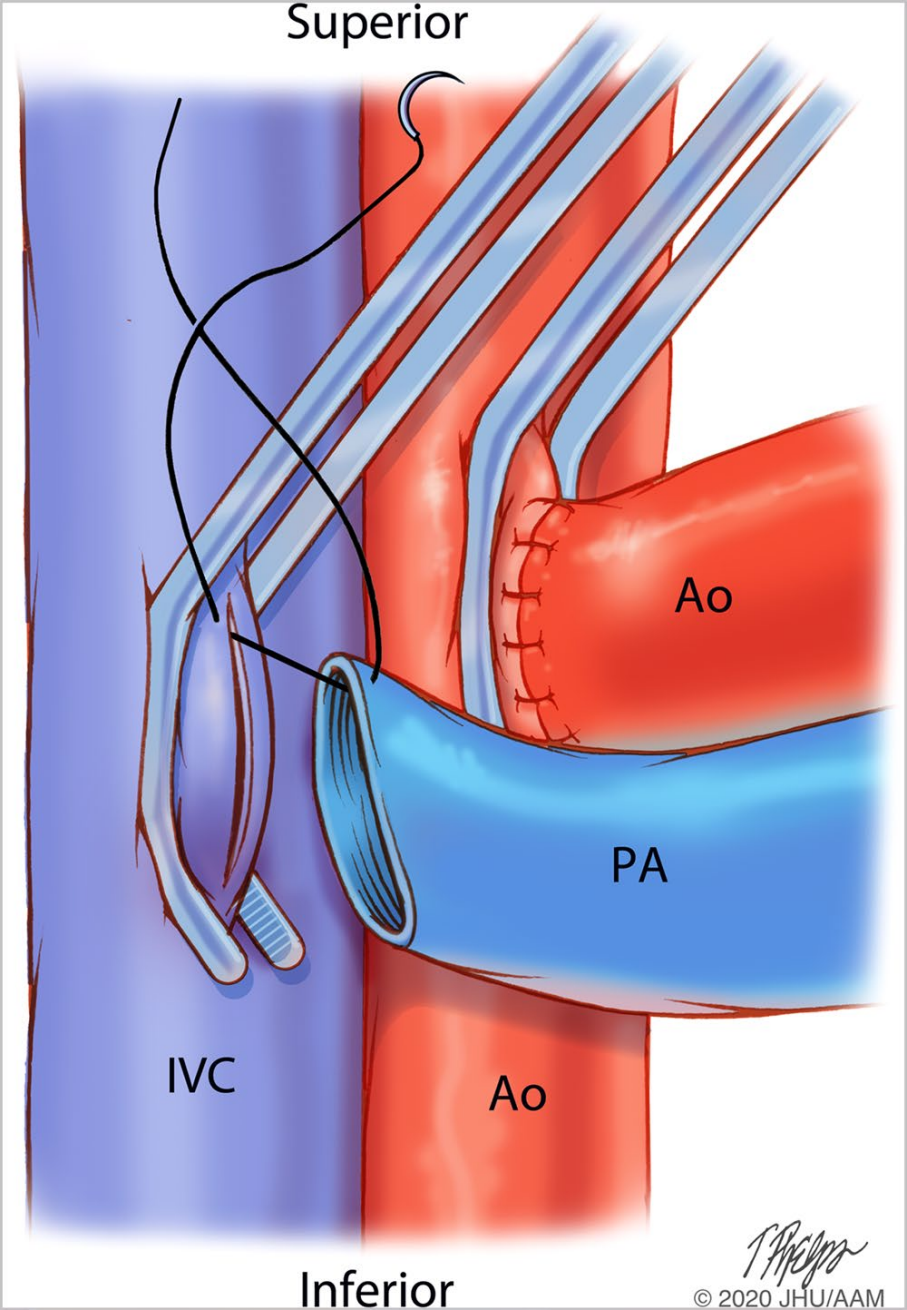
Aortic and Pulmonic Anastomosis to the Recipient. Abdomen. Image Copyright: Tim Phelps JHU/AAMM, 2020. Aortic and Pulmonic Anastomosis to the Recipient. Abdomen. Image Copyright: Tim Phelps JHU/AAMM, 2020.

Retroperitoneal exposure of the infrarenal abdominal aorta and IVC of the recipient is performed and proximal and distal control of these vessels are obtained. Aortotomy and cavotomies are placed approximately 2 centimeters (cm) distal to the renal vessel takeoffs. The aorta-aorta anastomosis is carried out before the pulmonic-caval anastomosis (Fig. 3).

Step-by-step performance of HHTx transplantation is as follows, with clinical pearls based on observation and experience denoted at relevant points of the procedure. All procedures described here have been approved by the Institutional Animal Care and Use Committee (IACUC) of our residing institution. Here we describe these steps in the context of pig-to-baboon xenotransplantation, however, this can be conducted similarly in baboon heterotopic allotransplantation.

#### Procurement of the Cardiac graft

##### Preparation of the cardiac donor

The donor is sedated with 10 milligrams per kilogram (mg/kg) of Ketamine intramuscular (I.M.) and 2 mg/kg xylazine for transfer to the operating room.

Clinical pearl*In swine, xylazine is used as an adjunct to ketamine, as many are somewhat refractory to the sedative effects of ketamine. While xylazine is known to cause second degree heart block and hypotension requiring atropine in some cases, this has not been our experience, although the true incidence is not known. Alternatively Ketamine and 0.12 mg/kg IV of Dexmedetomidine or Telazole 1 mg/kg can be used as an adjunct to ketamine*^1^.

A peripheral intravenous catheter is placed for medication administration. A 24 gauge angiocatheter in an ear or forelimb vein is preferred. Alternatively, a percutaneous femoral venous cannula can be placed if peripheral IV access is difficult to obtain. Additionally, a femoral arterial line is placed in either groin after appropriate sterility is obtained.

Induction of anesthesia is performed with isofluorene (1–1.5%). Routinely, both prophylactic amiodarone and lidocaine are given at 2 mg/kg and 1 mg/kg, respectively. A 6–7 mm cuffed endotracheal tube is used for oral tracheal intubation.

Anesthesia is maintained with isofluorene, with a goal minimal alveolar concentration (MAC) of 1.0–1.2. Paralytics such as succinylcholine and opiates such as fentanyl are given as needed to maintain paralysis and analgesia, respectively after ensuring appropriate levels of anesthesia.

Clinical pearl*In our experience, sevofluorene requires high doses for anesthetic effect in swine and thus the cardio suppressive effects of inhaled anesthetic agents is accentuated. However, there is some evidence that in the setting of experimentally induced MI models, sevoflurane increases the risk of fatal ventricular arrythmias. In regards to intubation, we sometimes elect to place a surgical tracheostomy as the swine anatomy can make oral tracheal intubation difficult and put the swine at risk for laryngospasms*^6,7^. *In terms of antiarrhythmics, lidocaine is well tolerated, however, amiodarone often causes bradycardia and hypotension. This can be mitigated by slow infusion, however, in severe cases this is refractory to most agents except calcium chloride, which can be given as a 100 mcg push. Amiodarone should be given only after hemodynamic stability is assured, an arterial line has already been placed and the surgeon is aware that it will be administered.*

Place EKG leads to the skin of the chest for cardiac monitoring, as lateral as possible to remain away from the surgical field.

Following intubation and induction of general anesthesia, the surgical field is prepped, with a series of three alternating scrubs with 10% betadine and 70% alcohol solution from the angle of the mandible to the upper abdomen at least 4 cm below the xyphoid process and laterally to the proximal arms.

##### Exposure of the donor cardiac graft

The skin is incised from above the sternal notch to below the xyphoid process using a 10-blade or cutting electrocautery. It is deepened through the subcutaneous fat and muscle using coagulating electrocautery. The sternal midline is identified by feeling the sternal edges in the rib interspaces and should be from associated muscle and tissue.

Clinical pearl*One must attend to the most superior portion of this incision. There is thick muscle to be cleared from the keel at the manubrium circumferentially and posteriorly.*

The xyphoid process may be amputated with electrocautery.

The retrosternal space is cleared from cardiac adhesions and pericardium using finger dissection and blunt scissors working superiorly from the subxyphoid space. Electrocautery may be used to take down diaphragmatic attachments.

An oscillating sternal saw or thick shears is used divide the sternum at the midline, taking care to protect the right ventricle from injury.

Clinical pearl*The manubrium’s keel is too thick to perform a midline sternotomy at this portion. Once resistance is met at the superior edge of the sternum, this area should be avoided and the sternum should be incised laterally in one direction or the other as the sternal saw is not able to cut this keel. Thus, soft tissue and muscle should be cleared here along this path as well. However, caution must be taken as the innominate vein is just deep to this area, as damage can cause devastating bleeding.*

Adequate hemostasis is ensured of the divided sternal edges utilizing electrocautery and bone wax if preferred.

An appropriately sized sternal retractor is placed for exposure and the pericardium is carefully divided. Take care to avoid irritation of the myocardium to prevent cardiac arrythmias. Pericardial sutures are placed with 2-0 silk to create a well, if needed.

A plane is created through sharp dissection or electrocautery between the ascending aorta and pulmonary artery to accommodate eventual cross-clamp of the aorta.

The superior vena cava is dissected inferior to the azygos vein and tagged with two 2-0 silk ties.

Clinical pearl*In swine, the azygos vein is small, posterior, and fragile. During dissection of the superior vena cava (SVC), care must be taken to avoid injuring or avulsing the azygos vein located on the posterior medial surface of the SVC. Alternatively, this can be ligated without consequence with surgical clips or tie.*

Similarly, the IVC is isolated and two silk ties are placed on a tension-free tag

##### Cardioplegia and arrest of donor cardiac graft

A purse-string or U-stitch of 5-0 Prolene is placed in the ascending aorta for securing of the cardioplegia cannula. This should be at least 2 cm distal to the aortic root. The cardioplegia cannula is placed and secured with a Rummel tourniquet.

Clinical pearl*Great care should be taken to prevent puncture of the posterior wall of the aortic root with the aortic cannula by pointing the tip of the needle bevel side anterior and pointed about 15 degrees inferiorly toward the aortic root. Hypotension can prevent proper resistance and distensibility of the aorta, increasing the risk of posterior wall puncture. Similarly, ensuring that systolic pressures are not higher than 120 mmHg prior to cannulation prevents excessive bleeding and reduces the risk of aortic dissection.*

500 un/kg of systemic heparin is administered. Ensure at least three minutes between administration and cross-clamp. Ensure the readiness of cardioplegia tubing and solution, sterile ice, and the entire team for cross-clamp and arrest.

In rapid sequence, ligate the cranial superior vena cava, place the aortic cross-clamp, vent the right atrium by dividing the inferior vena cava, and vent the left atrium either through incising the left atrial appendage or the inferior pulmonary veins. Immediately upon venting, begin running cardioplegia and fill the mediastinum with sterile ice slush.

Clinical pearl*It is important to prevent distention of the right and left ventricle with venting, as described above, for proper arrest and myocardial protection. Additionally, in order to facilitate optimal cooling of the heart on ice, the pleural spaces can be opened bilaterally allowing drainage of blood and cardioplegia. This prevents excess fluid from accumulating in the mediastinum and inefficient myocardial cooling.*

30 cc/kg of cardioplegia is administered through the cardioplegia cannula (Plegisol with 50 millimoles (mmol) sodium bicarbonate 8.4%, 50 mmol potassium chloride). Ensure that the heart is soft and relaxed with appropriate pressure (20 mmHg) and distention of the aortic root.

Clinical pearl*Cardioplegia should be kept on ice. Additionally, great care must be taken to ensure no air is present, as even small amounts of air can cause ischemia. Cardioplegia should be administered through pressure-resistant tubing (Table* *1* *for our preference) and a pressure bag. To ensure appropriate root pressures an aortic cannula arterial line can be placed, but this can be cumbersome. To a trained surgeon, this can be easily assessed by palpation of the aortic root.*

##### Donor cardiectomy

The superior vena cava is ligated and divided. The division of the inferior vena cava is completed from the venting step during cardioplegia administration, if necessary.

The aorta is incised distal to the cardioplegia cannula at the junction of the aortic root and arch. The pulmonary artery is incised just proximal to the bifurcation.

Cardiectomy is completed by dividing the remaining pulmonary veins along the pericardial reflection.

#### Backtable Preparation of the Cardiac Graft

##### Closure of caval orifices

The superior and inferior vena cava are tied with 2-0 silk prior to cardiectomy. However, alternatively this can be done on the backtable. (Fig. 2).

##### Closure of pulmonary vein orifices

A common orifice is created between the pulmonary veins at the level of the left atrium.

This common orifice is closed in two layers with a back-and-forth running 5-0 Prolene suture (Fig. 2).

##### Preparation of the aorta and pulmonary artery for anastomosis

The donor aorta and pulmonary should be beveled about 20–30 degrees from the axis parallel to the recipient IVC and abdominal aorta. It should also be an appropriate length to minimize kinking and tension (see Fig. 3). While this can be approximated on the backtable, it likely will need to be refined once the donor heart is placed in the recipient abdomen just prior to the anastomosis.

#### Exposure of the Recipient Abdominal Aorta

##### Preparation of the graft recipient

The recipient is sedated with 10 mg/kg of Ketamine I.M. for transport to the operating room.

Preoperatively, a tunneled triple lumen central venous catheter for administration of induction immunotherapy and maintenance immunotherapy, anticoagulation and other medications postoperatively is placed in either internal jugular veins (see Table 1 for model number). Our Immunosuppression has been previously described^1,3^.

One to two peripheral intravenous catheters for access for medication and fluid administration if needed and the central venous catheter is insufficient. 18–20 gauge catheters in forearm veins are preferred.

EKG leads are placed to the skin of the chest for cardiac monitoring, as lateral as possible to remain away from the surgical field.

A foley catheter is placed into the bladder for decompression and urine output monitoring

Following intubation and induction of general anesthesia, prepare the skin with a series of three alternating scrubs with 10% betadine and 70% alcohol solution from the xyphoid process to distal to the pubic symphysis across the bilateral groins for sterile femoral access.

Clinical pearl*We mirror our anesthesia strategy to the swine donor’s, as we believe the anesthesia strategy should mirror the cardioprotective approach.*

##### Safe entrance into the abdomen

A midline laparotomy is performed from the xyphoid to the pubic symphysis. The skin is incised using a 10 blade and deepened to the fascia using electrocautery.

Two forceps are used to elevate fascia and make a small (3 mm) sharp incision through fascia and peritoneum to enter the abdomen. Extend this incision to its full length using a finger or malleable retractor to protect the intra-abdominal contents (bowel) from transmitted electrocautery.

Clinical pearl*Great care should be taken to prevent electrocautery injury to the bowel. It is best to use Metzenbaum scissors for initial entry through the fascia into the abdomen. Additionally, the dome of the bladder should be cared for and avoided on the inferior portion of the incision. Despite a foley catheter, its peritoneal reflection can still be present above the pubic symphysis. While we have never encountered this, injuries to the bowel or bladder should be promptly repaired in 2 layers, the inner layer with an absorbable suture such as a 2-0 vicryl and a permanent suture such as a 2-0 silk for the outer layer. Missed bowel injuries can cause fatal abdominal sepsis in an immunosuppressed recipient.*

The bowel is protected with wet lap pad or towel and gently retracted cranially using an abdominal retractor.

The retroperitoneal space is opened inferior to the kidneys and at least 5 cm of inferior vena cava and abdominal aorta are exposed for accommodation of partially occlusive vascular clamps

The inferior vena cava and aorta are encircled with proximal and distal vessel loops, respectively, for proximal and distal control of the vessels.

#### Two-Anastomosis Implantation of the Cardiac Graft

##### Aorto-aortic anastomosis

A partially occlusive vascular clamp is placed on the superior portion of the infrarenal abdominal aorta after administration of 300 un/kg of heparin. See special notes for step 4.1.2.

The aortotomy is created sharply in a longitudinal fashion with an 11 blade, to a length of approximately 1 centimeter. Adjust this to accommodate the size of the donor ascending aorta with Pott’s scissors.

Clinical pearl*Take care not to cause a dissection of the abdominal aorta during this incision or injure the lateral or posterior walls during this incision. It is very important to remain partially occlusive on the abdominal aorta during the anastomosis to prevent prolonged totally occlusive clamp times. Additionally, variant anatomy sometimes yields an infrarenal abdominal aorta that is too small to accommodate an anastomosis and a suprarenal clamp must be placed instead. While this should be avoided, sometimes it is not possible but clamp times should be minimized to prevent kidney and spinal cord ischemia.*

An end-to-side, donor aorta to recipient infrarenal aorta, anastomosis using a running 5-0 Prolene on an RB-2 needle. Alternatively, the anastomosis can be created with two 5-0 prolene sutures as well.

Clinical pearl*The aortic anastomosis is performed prior to the pulmonary-caval anastomosis for technical reasons. We have found that the resultant orientation of the xenograft is more optimal and the sewing of the anastomosis is easier this way. If, when orienting the heart and creating the abdominal aortotomy and cavotomy, it is found to be more suitable to begin with the pulmonary anastomosis, one may do so. However, we have not found this to be the case.*

##### Pulmonary artery-caval anastomosis

A partially occlusive vascular clamp to the infrarenal IVC is placed after completion of the aorto-aortic anastomosis.

Clinical pearl*It is very important to remain partially occlusive during the anastomosis as prolonged totally occlusive clamp times of the IVC can contribute to bowel edema. See comments of vascular clamping noted in step 4.1.2.*

The inferior vena cava is opened sharply in a longitudinal fashion to a length of approximately 1 cm. It is adjusted to accommodate the size of the donor pulmonary artery remnant. The incision can be made using Pott’s scissors or an 11 blade.

Clinical pearl*Take care not to injure the lateral or posterior walls of the cava during this incision. Additionally, the cavotomy is usually placed slightly superior to the aortotomy’s position as to accommodate proper placement of the heart in the abdomen to prevent kinking or tension*.

The xenograft is placed in a position that will not cause tension on the aorto-aortic anastomosis and trim the donor pulmonary artery to the shortest possible length to create an end-to-side caval anastomosis in this position.

An end-to-side, donor pulmonary artery to recipient IVC, anastomosis is performed using a running 5-0 Prolene on an RB-2 needle. The xenograft can be temporarily flipped laterally over the axis of the anastomosis in order to expose the contralateral edge of the anastomosis if needed. Alternatively, similar to the arterial anastomosis, two 5-0 Prolene anastomosis can be performed, depending on surgeon preference.

Partially occlusive vascular clamps can be placed superiorly or inferiorly oriented, depending on exposure, angle and orientation of the cardiac graft (Fig. 3)

#### Reperfusion of the cardiac graft

An 18-gauge angiocath needle is used to access the apex of the left ventricle for de-airing. This site will ultimately be used for implantation of the telemetry pressure probe.

Administer systemic sodium bicarbonate 1 mmol/kg, lidocaine 1 mg/kg, and mannitol 0.3 gm/kg in preparation for reperfusion.

The IVC clamp is removed first to allow for retrograde filling of the right ventricle, followed by release of the aortic cross clamp.

Clinical pearl*Be prepared for electric cardioversion in the case of fibrillation. Hearts of swine are prone to ventricular arrhythmias and is very common upon reperfusion. Prompt unsynchronized internal fibrillation of 10–15 Joules should be initiated.*

#### Implantation of the telemetry device

A pocket is formed in the lateral abdominal wall, between the fascia and muscle of the external oblique, to accommodate a telemetry device.

The telemetry device is placed in this pocket and the fascia closed over the device, for soft tissue coverage of the device, prior to implanting the pressure probe and EKG leads.

The pressure probe is placed in the apex of the left ventricle, preferably at the prior site of de-airing. Secure the probe with a 5-0 Prolene purse string using felt pledgets.

Clinical pearl*Notably, some telemetry devices come with two pressure probe channels. The pressure probes can be safely placed in the left or right atrial appendage or ascending aorta, if other parameters are desired.*

The first EKG lead to the left ventricle is placed with a simple 5-0 Prolene stitch. The second lead may be free in the peritoneal cavity as a ground.

#### Closure of the Recipient Abdomen

The midline fascia is closed with running 0 Prolene suture. Take care to avoid catching the leads in the closure and protect bowel during closure.

Close the remaining incision in two layers using absorbable suture (2-0 Vicryl followed by 3-0 vicryl at the skin is our preference).

Clean and dry the incision and apply skin glue. Dress the wound as desired for 48 hours.

Recover the animal from general anesthesia.

Place on systemic anticoagulation (e.g., heparin) 24 hours after surgery, once surgical risk is deemed minimal. The goal ACT is twice the level of baseline or aPTT 60–80.

#### Transabdominal Myocardial Biopsies

The recipient is placed under anesthesia as previously described, along with administration of prophylactic antiarrhythmics.

The point of maximal cardiac impulse is located and the smallest incision at the skin and underlying fascia, as required to safely perform a myocardial punch biopsy, is performed to expose the heart.

Any scar tissue, overlying bowel or omentum is cleared from the heart and a point for biopsy is located.

Similar for telemetry pressure lead placement (as described above), a 5-0 Prolene purse string using felt pledgets is performed around the site of planned punch biopsy

A full thickness punch biopsy is performed using a 2-3 Fr punch biopsy tool at the center of this purse string

The purse string is tied down in a standard fashion and hemostasis is ensured

Closure of the abdominal wall is closed as previously described above

### Long-term evaluation of the cardiac graft

The primary mechanisms for evaluation of results are the following: transabdominal palpation of the xenograft, transabdominal ultrasonography of the xenograft, direct vascular, atrial or ventricular pressure and EKG monitoring utilizing the implanted telemetry device and serial serum laboratory values including complete blood count and troponin. Transabdominal myocardial punch biopsies are performed a maximum of two times over the lifetime of the recipient, at least one month apart, as limited by our local IACUC for histopathologic surveillance (described in detail above).

**Figure 4:**
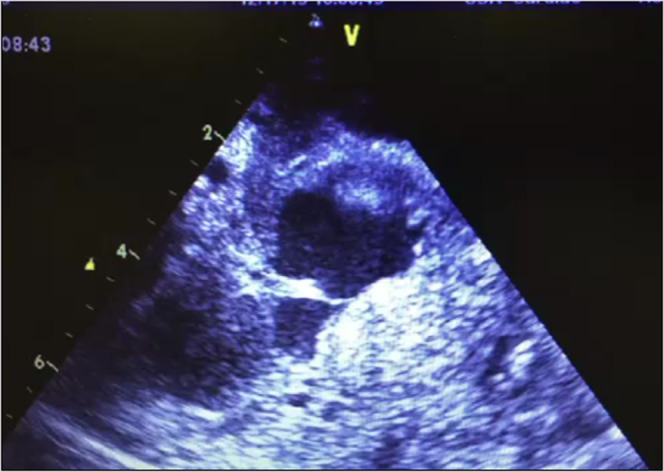
Left ventricular thrombus in porcine xenograft.

Palpation allows the easy grading by feel of the contractility of the xenograft. A system of 0 to 4+ is utilized, where 0 indicates no contractility and 4+ indicates full contractility (supplementary video 2). On transabdominal ultrasonography, the contractility of the left and right ventricles, the presence or absence of left ventricular thrombus and/or left ventricular hypertrophy may be assessed (supplementary video 2). Left ventricular thrombus (Fig. 4) is common but may be partially or completely resolved with the administration of a systemic heparin infusion, which is recommended to be performed routinely.

Progressive left ventricular hypertrophy may indicate ongoing xenograft rejection and will generally be seen prior to depressed left ventricular function. Elevated and increasing troponin, particularly in conjunction with thrombocytopenia, can increase the suspicion for rejection. The most precise measure, however, is pressure tracings of the telemetry device, which we have previously characterized^8^. We have found the most sensitive measure of rejection is an increase in left ventricular end diastolic pressure (LVEDP). Cardiac contractility ceases after the pulse pressure is reduced to <10 mmHg. We have also previously characterized intracardiac thrombus as an indicator of rejection (Fig. 4)^4^.

## Results and Discussion

We have extensive experience with this model over the last 10 years. With establishment of this model and consistent long-term survival, we can now take observations made *in vitro* that can be rigorously tested. Additionally, this model can yield clinical insights regarding allotransplantation, immunology and cardiac specific tissue injury. We have been able to characterize and expand CD4 + CD25 + FoxP3+ regulatory (Treg) T-cells and demonstrate their suppressive effects onto xenografts, recipient B and T-cell populations and their potential role in allotransplantation^9–11^. Additionally, we have shown that Rapamycin, a currently clinically approved immunosuppressive drug in allotransplantation, promotes enrichment of functional Treg cells with immunoregulatory properties^12^. Lastly, we have extensively characterized transgenic pigs for the use in cardiac xenotransplantation and identified early markers for rejection that are applicable to not only cardiac xenotransplantation but also as a generic marker of tissue injury relevant to other fields of study and are graft specific for this model^13–16^.

Lastly, we have extensively studied co-stimulation blockade and B-cell depletion’s role in xenotransplantation, which has transformed the field and extended survival not only in cardiac, but also kidney, liver and islet cell xenotransplantation^17–19^.

There are several critical steps in this procedure. Smooth procurement of the xenograft, with adequate myocardial protection, is the first critical step. Anastomosis in the recipient abdomen in a way that avoids narrowing or kinking of either of the two anastomoses, but particularly the pulmonary artery-caval anastomosis, is the next. Finally, maintenance of xenograft contractility in a normal sinus rhythm is necessary for coronary perfusion and ultimately xenograft survival.

During procurement of the xenograft, the cardioplegia must be administered under pressure. The heart must be adequately vented and the output should become clear. The distention of the aortic root can be assessed manually, as can the distention of the xenograft itself. Generally, if there is difficulty running the cardioplegia or distention of the graft or root, extending the incision in the inferior vena cava, left atrial appendage, and/or pulmonary veins will aid in venting and resolve this difficulty. Once transferred into the recipient abdomen, care in the performance of the anastomoses, such that the vascular lumens to not become narrowed, is critical. The geometry of the xenograft can be assessed during implantation. The more common error in this step is creating a pulmonary-artery caval connection in which the pulmonary artery remnant is too long, and allows the xenograft to fold on itself and kink the anastomosis. Finally, utilization of both chemical and electrical means to establish normal sinus rhythm early following implantation is imperative.

**Figure 5:**
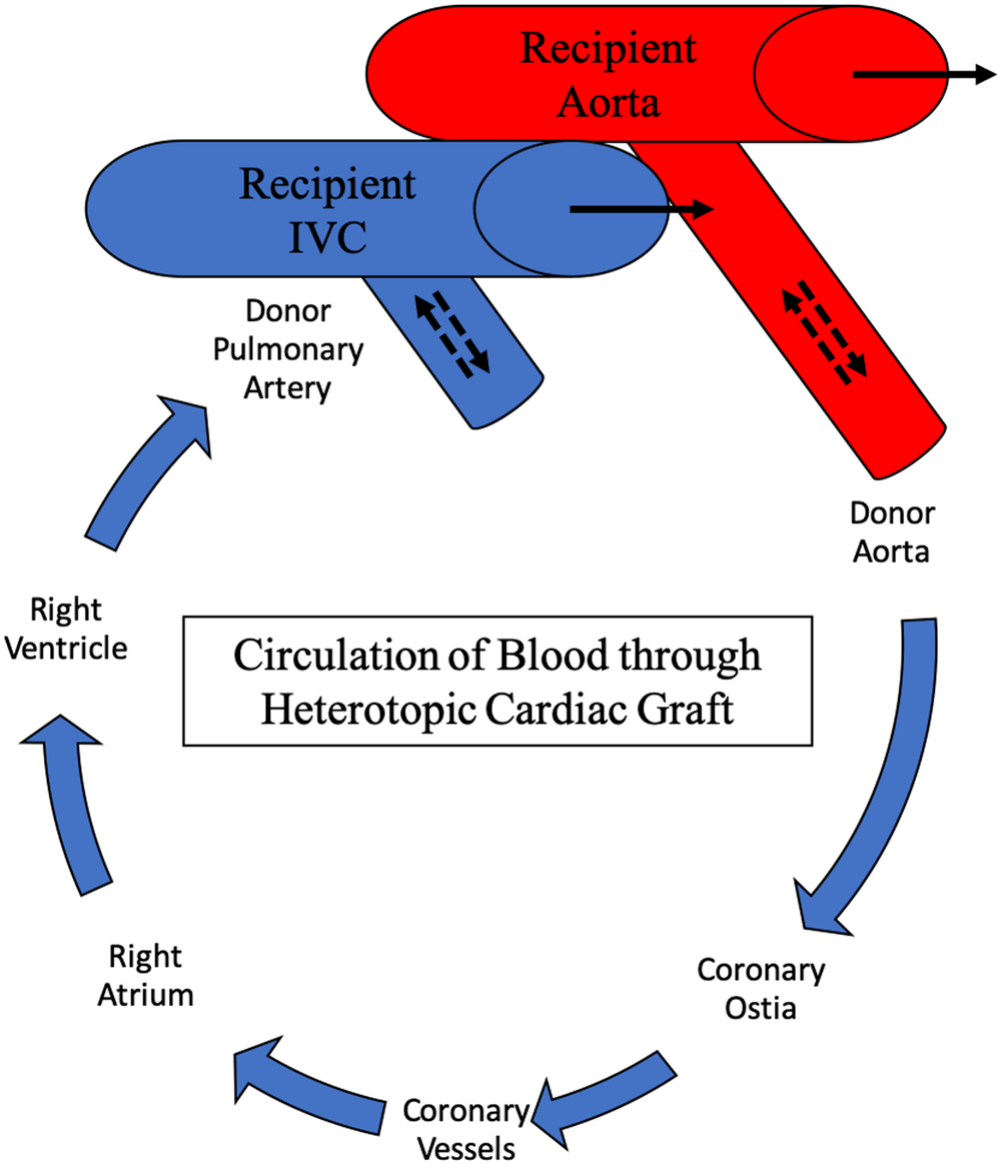
Perfusion of Blood Through the Heterotopic Cardiac Graft: The graft is perfused via its coronary vessels during graft diastole and aortic valve cooptation, supplied from the recipient aorta. Perfused blood from the graft is then drained into the right atrium via the coronary sinus. Solid arrows denote recipient native blood flow. Dotted arrows represent graft blood flow in systole (outward from graft major vessels) and diastole (into graft atria/ventricles).

The limitations of this model are in its non-life-supporting nature. The native recipient heart supplies complete cardiac output, hemodynamic support and end-organ perfusion. The intra-abdominal xenograft does not contribute to this support. Rather, it remains in continuity with the recipient’s vascular supply, allowing continuous perfusion of the xenograft with recipient blood and exposure to the full recipient immune response (Fig. 5).

However, this non-life supporting model allows the evaluation of cardiac transplant rejection, testing of different porcine genetic modifications and immunosuppressive regimens, without life-threatening physiologic perturbations of the recipient. Life-sustaining studies require the recipient to be placed on cardiopulmonary bypass just to undertake the transplantation. Additionally, these recipients require critical care postoperatively, as perturbations of the cardiac graft can compromise supporting perfusion for all vital organs. This model requires no critical care and can be immediately transferred to a non-intensive setting similar to preoperative housing.

The incidence of cardiovascular disease causing end-stage heart failure requiring transplantation is growing^20^. Genetically modified porcine organs that have been tested in this model and that are now being used in orthotopic studies are increasingly meeting preclinical efficacy requirements for their eventual use^21–23^. While survival has been obtained for up to 6 months, there is much still to learn regarding the physiologic and immunologic mismatch between swine and baboon (and human for that matter) as these hearts eventually still succumb to end-stage failure by an unknown mechanism^24–27^. Additionally, porcine endogenous retrovirus (PERV) knockout swine have been produced out of concern for the theoretical transmission of PERV (although there has been no evidence of human transmission and PervC null animals are being produced regularly without gene editing)^28–31^.

All things considered, this model continues to be relevant for demonstrating pre-clinical efficacy, safety and testing iterative changes in genetic constructs of porcine xenografts. Further successes in reproducing this model in other institutions may enable continued enhancement of porcine cardiac xenotransplantation for the eventual use in humans, but also push the boundaries of discovery as has been limited by other disease models across disciplines. For example, knowledge gained by insights in xenotransplantation, an arduous immunological barrier in excess of human allotransplantation, can shed light onto strategies to abrogate problems faced by dysregulation in immunity such as antibody mediated rejection or graft versus host disease. Additionally, as this model uniquely provides *in vivo* assessment of whole organ function without compromising host physiology, it serves as a way to assess cardiac physiology beyond the limitations of what current models can provide^32–33^.

## Supplementary information

Supplementary Video 1.

Supplementary Video 2.
